# Comparative Evaluation of the Antidiabetic, Hypolipidemic and Antioxidant Effects of *Polygonum persicaria* L. Herb and *Vaccinium myrtillus* L. Leaves in Streptozotocin-Induced Diabetes

**DOI:** 10.3390/molecules31122080

**Published:** 2026-06-13

**Authors:** Kostici Roxana, Pirscoveanu Denisa Floriana Vasilica, Diana-Maria Trasca, Adina Maria Kamal, Carmen Vladulescu, Renata Maria Varut, Pluta Ion Dorin, Daniela Cîrțînă, Maria Stoica, Romeo Popa, Gabriela Pura

**Affiliations:** 1 Department of Toxicology, Faculty of Pharmacy, University of Medicine and Pharmacy of Craiova, 2 Petru Rareş Street, 200349 Craiova, Romania; roxana.kostici@umfcv.ro; 2 Department of Neurology, Faculty of Medicine, University of Medicine and Pharmacy of Craiova, 200349 Craiova, Romania; denisa.pirscoveanu@umfcv.ro; 3 Department of Internal Medicine, University of Medicine and Pharmacy of Craiova, 200349 Craiova, Romania; diana.trasca@umfcv.ro (D.-M.T.); adina.kamal@umfcv.ro (A.M.K.); 4 Faculty of Horticulture, Department of Biology and Environmental Engineering, University of Craiova, 200585 Craiova, Romania; carmen.vladulescu@edu.ucv.ro; 5 Research Methodology Department, Faculty of Pharmacy, University of Medicine and Pharmacy of Craiova, 200349 Craiova, Romania; 6 Faculty of Medical and Behavioral Sciences, Constantin Brâncuși University of Târgu Jiu, 210185 Târgu Jiu, Romania; dorin.pluta@e-ucb.ro; 7 Department of Intensive Care and Anesthesia, Emergency County Hospital, 200349 Craiova, Romania; maria.stoica@umfcv.ro; 8 Department of Pharmacology, University of Medicine and Pharmacy of Craiova, 200349 Craiova, Romania; romeo.popa@umfcv.ro; 9 Department of Medical Devices and Pharmaceutical Practice, Iuliu Hațieganu University of Medicine and Pharmacy, 400012 Cluj-Napoca, Romania; gabrielapura@gmail.com

**Keywords:** diabetes mellitus, *Polygonum persicaria*, *Vaccinium myrtillus*, oxidative stress, polyphenols, hypoglycemic activity, lipid metabolism, antioxidant enzymes

## Abstract

**Background/Objectives:** Diabetes mellitus is a chronic metabolic disorder characterized by hyperglycemia, dyslipidemia, and oxidative stress, leading to severe systemic complications. Medicinal plants rich in polyphenolic compounds have gained increasing attention as complementary therapeutic agents. This study aimed to comparatively evaluate the chemical composition, as well as the antidiabetic, hypolipidemic, and antioxidant effects of *Polygonum persicaria* and *Vaccinium myrtillus* in a streptozotocin-induced diabetic model. Although *Vaccinium myrtillus* has been more extensively investigated for its antidiabetic potential, the pharmacological relevance of *Polygonum persicaria* in diabetes remains insufficiently characterized, particularly in direct comparison with a recognized phytotherapeutic comparator. **Methods:** Hydroalcoholic tinctures prepared from *Polygonum persicaria* L. herb and *Vaccinium myrtillus* L. leaves were subjected to phytochemical analysis using High-Performance Thin-Layer Chromatography (HPTLC) for the identification of flavonoids and phenolcarboxylic acids, alongside spectrophotometric determination of total polyphenol and flavonoid content. Experimental diabetes was induced in CD1 mice by streptozotocin administration. Animals were treated orally for 35 days, and glycemic parameters, lipid profile, body weight, food and water intake, and oxidative stress markers (MDA, SOD, TAC, and GPx) were evaluated. **Results:** HPTLC/CSS screening indicated the presence of rutin, chlorogenic acid, and caffeic acid in *Polygonum persicaria*, while *Vaccinium myrtillus* showed stronger densitometric signals for phenolcarboxylic acid-type compounds, particularly chlorogenic and caffeic acids. Total polyphenol and flavonoid content were also higher in *Vaccinium myrtillus* (433.89 ± 8.67 mg/L GAE; 154.38 ± 3.08 mg/L QE) compared to *Polygonum persicaria* (269.28 ± 5.25 mg/L GAE; 132.75 ± 2.65 mg/L QE). Functionally, *Vaccinium myrtillus* demonstrated a significant antihyperglycemic effect from day 14 (*p* = 0.009) and improved lipid parameters, while *Polygonum persicaria* showed a delayed glycemic effect, significant only at day 35 (*p* = 0.014), without significant hypolipidemic activity. In contrast, *Polygonum persicaria* exerted a marked antioxidant effect, significantly increasing GPx activity (*p* = 0.025) and reducing MDA levels (*p* = 0.053). **Conclusions:** *Vaccinium myrtillus* showed stronger antihyperglycemic and hypolipidemic effects, while *Polygonum persicaria* was mainly associated with antioxidant-related biochemical changes. These differences may be influenced by phytochemical composition, but they cannot be attributed solely to total polyphenol or flavonoid content.

## 1. Introduction

Diabetes mellitus (DM) is a multifactorial chronic metabolic disorder characterized by persistent hyperglycemia resulting from impaired insulin secretion, insulin action, or both [1]. It represents one of the most significant global health challenges of the 21st century, with a continuously increasing prevalence driven by population aging, urbanization, sedentary lifestyle, and the rising incidence of obesity [2]. The International Diabetes Federation has highlighted the alarming magnitude of this condition, reporting hundreds of millions of affected individuals worldwide, many of whom remain undiagnosed.

Beyond hyperglycemia, diabetes is associated with profound disturbances in carbohydrate, lipid, and protein metabolism, leading to a complex metabolic syndrome with severe systemic consequences [3]. Chronic hyperglycemia contributes to long-term damage and dysfunction of multiple organs, including the eyes, kidneys, nervous system, and cardiovascular system, ultimately increasing morbidity and mortality [4,5,6,7]. In particular, type 2 diabetes mellitus (T2DM), the most prevalent form, is characterized by a progressive decline in pancreatic β-cell function combined with insulin resistance in peripheral tissues, influenced by both genetic predisposition and environmental factors such as diet and physical inactivity [8].

An essential pathogenic mechanism linking hyperglycemia to diabetic complications is oxidative stress. Persistent elevation of blood glucose levels promotes the overproduction of reactive oxygen species (ROS) through several biochemical pathways, including glucose auto-oxidation, protein glycation with formation of advanced glycation end products (AGEs), and mitochondrial dysfunction [9]. In addition, activation of the polyol pathway and protein kinase C isoforms contributes to NADPH depletion, altered redox homeostasis, and further ROS generation [10]. These mechanisms are considered central links between chronic hyperglycemia, oxidative stress, endothelial dysfunction, and the development of diabetic complications [11].

Oxidative stress is also amplified by glucose auto-oxidation and protein glycation, which impair antioxidant defense mechanisms and enhance free radical production. The antioxidant defense system consists of endogenous components, such as superoxide dismutase (SOD), total antioxidant capacity (TAC), and glutathione peroxidase (GPx), as well as exogenous antioxidants, including ascorbate, bioflavonoids, carotenoids, and tocopherols. This imbalance between pro-oxidant and antioxidant systems leads to lipid peroxidation, protein oxidation, DNA damage, and activation of inflammatory signaling pathways, thereby contributing to the development of diabetic complications such as nephropathy, neuropathy, retinopathy, and cardiovascular disease. Moreover, oxidative stress contributes to β-cell dysfunction and apoptosis, further aggravating insulin deficiency and metabolic dysregulation [12,13].

In addition to oxidative stress, dyslipidemia is a common feature of diabetes, characterized by elevated levels of total cholesterol, triglycerides, and low-density lipoproteins, along with decreased high-density lipoproteins. These lipid abnormalities significantly increase the risk of atherosclerosis and cardiovascular complications, which represent the leading cause of death among diabetic patients. Therefore, therapeutic strategies targeting not only glycemic control but also lipid metabolism and oxidative stress are essential for comprehensive diabetes management [14,15].

Given the limitations of conventional pharmacological therapies, including adverse effects and limited efficacy in preventing complications, increasing attention has been directed toward the use of medicinal plants as alternative or complementary therapeutic agents. Ethnopharmacological data indicate that more than 1200 plant species exhibit hypoglycemic activity [16], many of them containing bioactive compounds such as flavonoids, phenolic acids, alkaloids, terpenoids, and glycosides with proven antidiabetic potential [17,18]. These compounds exert their effects through multiple mechanisms, including stimulation of insulin secretion, enhancement of insulin sensitivity, inhibition of intestinal glucose absorption, modulation of glucose transporter expression (e.g., GLUT4), activation of nuclear receptors such as PPARγ, and regulation of key metabolic enzymes. In addition, metabolites such as phenolic acids, phenylpropanoids, and terpenoids exhibit significant antioxidant properties, supporting their role in mitigating oxidative stress associated with diabetes [19].

*Vaccinium myrtillus* L. leaves (*Myrtilli folium*, MF) have been traditionally used as a vegetal product with potential antidiabetic relevance. The leaves are rich in polyphenolic constituents, including flavonoids, phenolic acids, and tannin-type compounds, which have been associated with antioxidant, anti-inflammatory, hypoglycemic, and hypolipidemic effects [20,21]. Previous experimental studies have reported that preparations obtained from V. myrtillus may improve glycemic control, reduce oxidative stress, and modulate lipid metabolism in diabetic models [22,23,24]. In the present study, V. myrtillus leaf tincture was used as a positive phytotherapeutic comparator because of its documented antidiabetic potential and because preliminary doctoral research identified it as an active reference preparation in streptozotocin-induced diabetes [25].

In contrast, *Polygonum persicaria* L. herb (*Polygoni persicariae herba*, PPH; syn. *Persicaria maculosa*) is a less explored medicinal plant, despite its traditional use and the presence of numerous bioactive constituents. Previous phytochemical investigations have identified a complex composition including carbohydrate derivatives (erythritol, glycerol, xylitol, D-fructose), phenolic compounds, isoflavones, phytosterols, and fatty acids such as palmitic, stearic, and linoleic acids [26]. Furthermore, studies have reported antifungal [27] and insecticidal [28] activities, suggesting a broad spectrum of biological effects. Traditional data also indicate potential hypoglycemic and antidiarrheal properties [29]; however, its role in diabetes management, particularly regarding lipid metabolism and oxidative stress modulation, remains insufficiently investigated.

Considering the complex pathophysiology of diabetes, involving hyperglycemia, dyslipidemia, and oxidative stress, the identification of plant-derived compounds with multitarget effects is of considerable interest. Comparative studies between well-established medicinal plants such as *Vaccinium myrtillus* and less studied species like *Polygonum persicaria* may provide valuable insights into new therapeutic options.

In this context, the antioxidant potential of the studied plant extracts was evaluated in a streptozotocin-induced chronic diabetic model. After five weeks of administration, oxidative stress parameters were assessed, including malondialdehyde (MDA, determined by ELISA, ng/mL), superoxide dismutase (SOD, U/mL), and glutathione peroxidase (GPx, U/L in hemolyzed blood). These parameters were compared with those of the control group, the untreated diabetic group, and the group treated with *Vaccinium myrtillus* extract, used as a positive control due to its established antidiabetic effects.

Therefore, the aim of the present study is to investigate the chemical composition and to evaluate the antidiabetic, hypolipidemic, and antioxidant effects of *Polygonum persicaria* in comparison with *Vaccinium myrtillus*, using experimental models of streptozotocin-induced diabetes. The study focuses on the assessment of glycemic parameters, lipid profile, oxidative stress biomarkers, and histopathological changes, in order to elucidate the potential mechanisms underlying their biological activity and to support their possible use as complementary agents in diabetes management. Therefore, the present study addresses an important gap by directly comparing the phytochemical profile and biological effects of PPH and VMF in the same streptozotocin-induced diabetic model.

## 2. Results

The results are presented in a sequential manner, beginning with the phytochemical characterization of the selected tinctures, followed by the evaluation of acute toxicity, glycemic evolution, lipid profile, food and water intake, oxidative stress parameters, and histopathological findings. This organization allows an integrated comparison between PPH and VMF in terms of chemical composition and biological activity.

### 2.1. Phytochemical Analysis of Selected Tinctures (HPTLC)

Based on comparison with available reference standards, HPTLC analysis indicated the presence of flavonoids and phenolcarboxylic acids in the investigated tinctures of MF and PPH, with several components detected as characteristic chromatographic bands (Table 1 and Table 2). In situ UV spectral comparison was used as an additional criterion to support tentative marker identification. The UV spectra of chromatographic bands detected in the tinctures were compared with those of available reference standards, together with Rf values and densitometric profiles. Therefore, UV analysis was not used as an independent quantitative method, but as a complementary tool for chromatographic band assignment.

Regarding flavonoids, a chromatographic band corresponding to rutin was detected in the *Polygonum persicaria* tincture, based on Rf value and in situ UV spectral comparison with the rutin reference standard. Although flavonoid-type bands were also observed in the *Vaccinium myrtillus* tincture, their precise assignment was not possible because appropriate reference standards for all detected flavonoids were not available.

**Figure 1:**
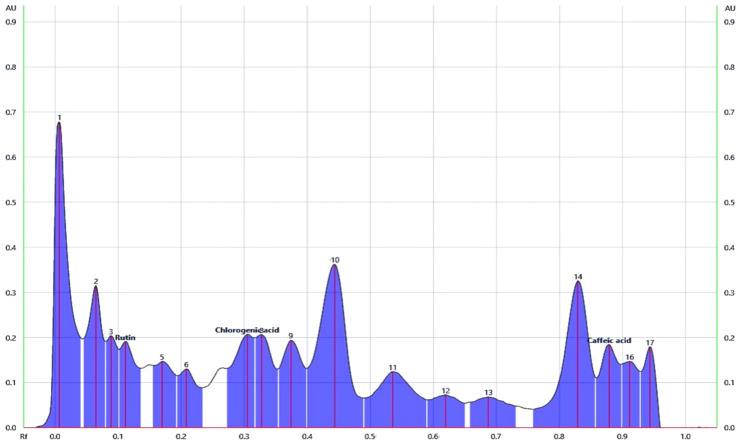
Densitogram of polyphenols separated from the PPH tincture (UV λ 280 nm, without derivatization). PPH: *Polygoni persicariae herba*.

**Figure 2:**
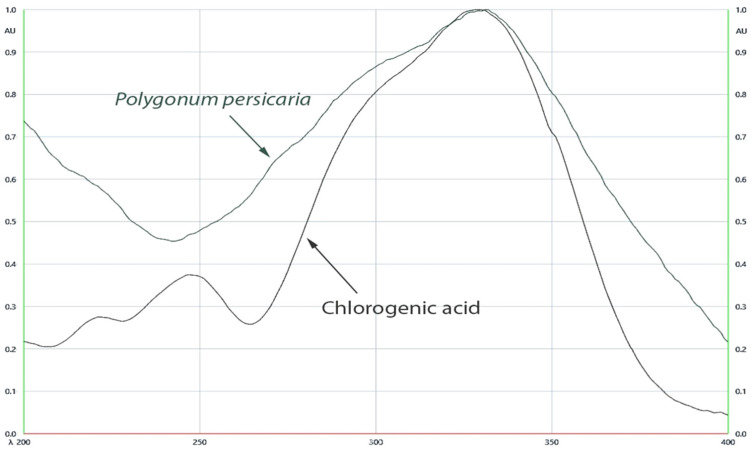
In situ UV spectrum of the chlorogenic acid standard compared with chlorogenic acid separated from the PPH tincture (UV λ 280 nm, without derivatization). PPH: *Polygoni persicariae herba*.

**Figure 3:**
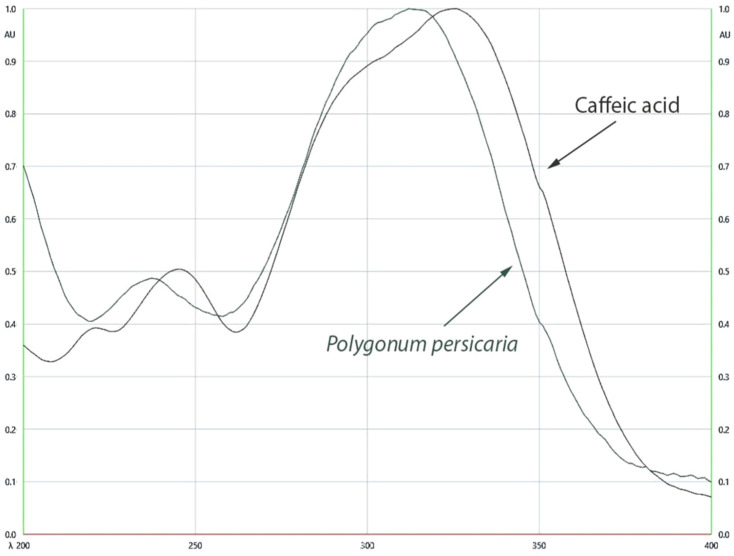
In situ UV spectral comparison between the caffeic acid reference standard and the corresponding chromatographic band detected in the PPH tincture, supporting tentative identification based on spectral similarity and chromatographic behavior.

**Figure 4:**
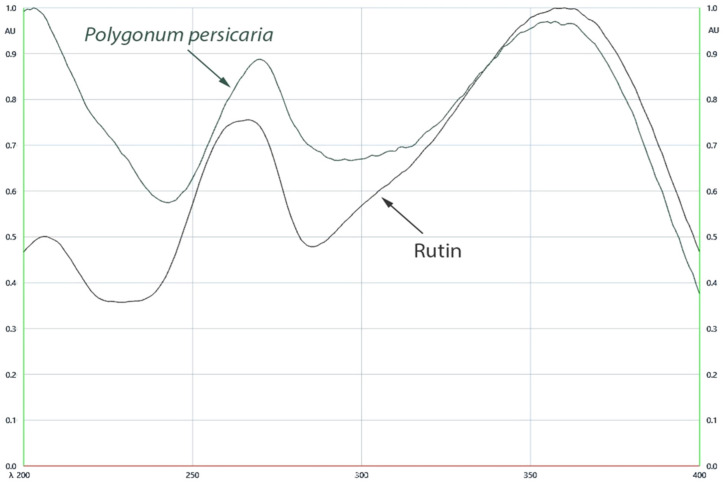
In situ UV spectral comparison between the rutin reference standard and the corresponding chromatographic band detected in the PPH tincture.

**Figure 5:**
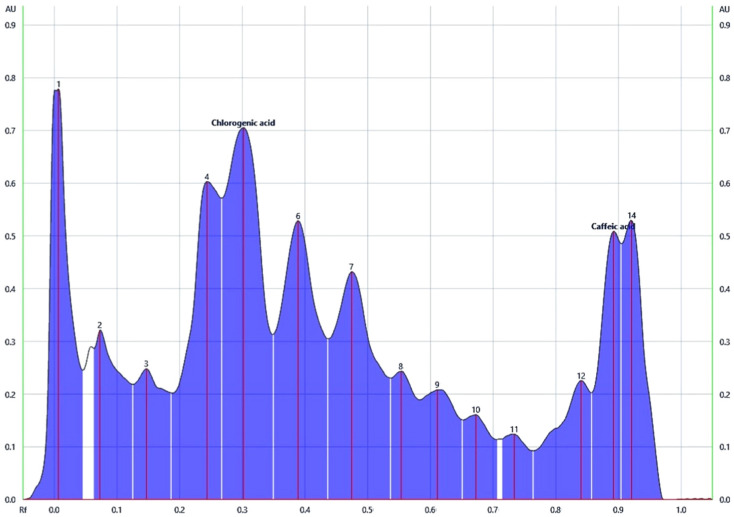
HPTLC densitogram of polyphenolic compounds separated from the MF tincture recorded at UV λ 280 nm, without derivatization.

**Figure 6:**
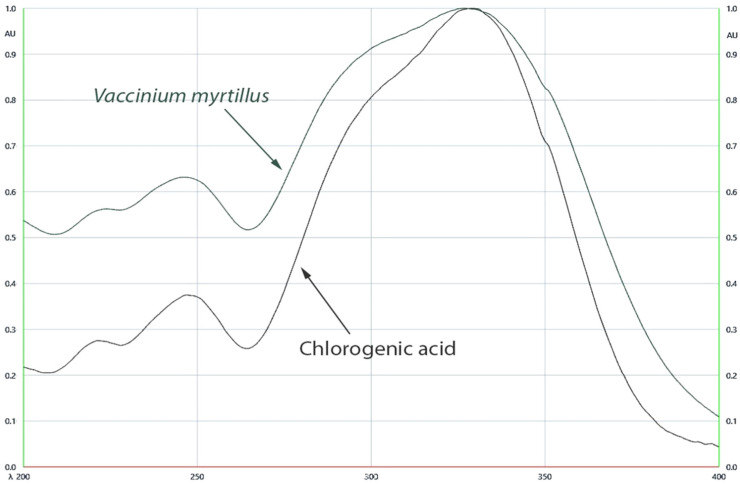
In situ UV spectral comparison between the chlorogenic acid reference standard and the corresponding chromatographic band detected in the MF tincture.

**Figure 7:**
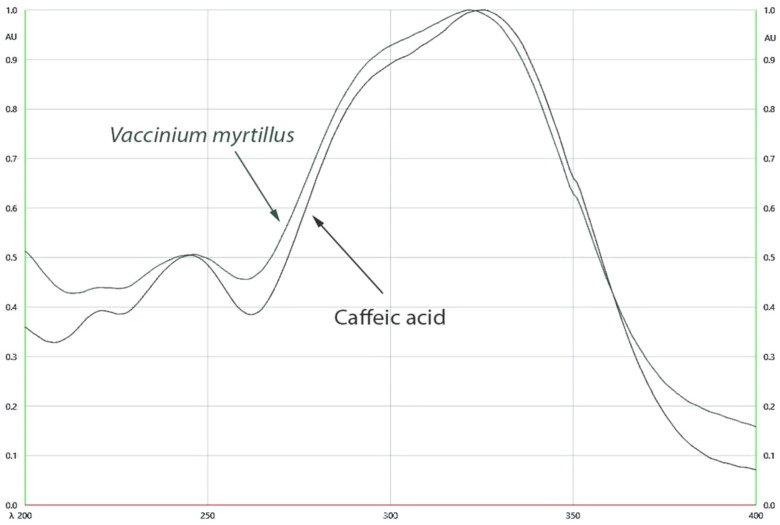
In situ UV spectral comparison between the caffeic acid reference standard and the corresponding chromatographic band detected in the MF tincture.

In terms of phenolcarboxylic acids, chromatographic bands corresponding to chlorogenic acid and caffeic acid were detected in both tinctures, based on comparison with the available reference standards. The densitometric response suggested a higher relative abundance of chlorogenic acid- and caffeic acid-type compounds in *Vaccinium myrtillus* compared with *Polygonum persicaria* (Figure 1, Figure 2, Figure 3, Figure 4, Figure 5, Figure 6 and Figure 7). Additional phenolcarboxylic acid-type bands were also observed; however, their exact identification was not possible due to the lack of corresponding standards.

These findings are consistent with literature data reporting a rich polyphenolic profile for both *Vaccinium myrtillus* [30] and *Polygonum persicaria* [31]. However, because the HPTLC method was not fully validated for definitive compound-specific quantification and no internal standard was used, the densitometric data should be interpreted only as semi-quantitative comparative estimates. Therefore, the HPTLC analysis was used primarily for phytochemical screening and tentative marker identification rather than for precise quantitative determination.

The densitometric values are reported as semi-quantitative comparative estimates only, because the HPTLC method was not fully validated for compound-specific quantitative determination and no internal standard was used.

### 2.2. Total Polyphenol and Flavonoid Content

The total polyphenol content, expressed as mg/L gallic acid equivalents (GAE), differed between the two analyzed tinctures. Among the investigated samples, MF tincture exhibited the highest polyphenol content, with a value of 433.89 ± 8.67 mg/L GAE, whereas PPH tincture showed a lower value of 269.28 ± 5.25 mg/L GAE.

A similar trend was observed for total flavonoid content, expressed as mg/L quercetin equivalents (QE). MF showed higher flavonoid levels, 154.38 ± 3.08 mg/L QE, compared with PPH, 132.75 ± 2.65 mg/L QE (Table 3).

### 2.3. Acute Toxicity

No mortality was recorded after oral administration of *Polygonum persicaria* L. herb tincture at doses ranging from 1 to 9 g/kg body weight. At doses of 2–5 g/kg, transient somnolence and tachycardia were observed approximately 10 min after administration, with recovery within 2 h. At higher doses of 6–9 g/kg, sedation was also observed shortly after administration, but normal behavior was restored within 24 h. A slight decrease in body weight was recorded after 24 h, while blood glucose showed variable changes. Since no mortality occurred at the highest tested dose, the oral LD50 was considered to be >9 g/kg body weight, indicating low acute toxicity under the present experimental conditions (Table 4).

The acute toxicity results showed that the hydroalcoholic tincture of *Polygonum persicaria* L. herb was well tolerated after oral administration, with no mortality observed up to the highest tested dose of 9 g/kg body weight. Based on the absence of lethality and on the estimated oral LD50 value exceeding 9 g/kg body weight, the preparation showed a wide acute safety margin. According to the classification of acute systemic toxicity based on oral LD50 values [32], this estimated value is consistent with toxicity class 5, corresponding to the lowest acute toxicity category.

### 2.4. Oral Glucose Tolerance Test

In the preliminary oral glucose tolerance test, three doses of each tincture were evaluated: 100, 150, and 200 mg/kg body weight. The dose range of 100, 150, and 200 mg/kg body weight was selected as a preliminary screening interval based on tolerability, available extract quantity, and previous exploratory pharmacological observations. Although the tested doses were relatively close, they were intended to identify a suitable working dose for the chronic experiment rather than to establish a complete dose–response relationship. For *Polygonum persicaria* L. herb tincture, the dose of 200 mg/kg body weight produced the most consistent glucose-lowering effect, with significantly lower glucose values at 90 and 120 min after glucose administration. For *Vaccinium myrtillus* L. leaf tincture, the 100 and 150 mg/kg doses showed statistically significant reductions at 120 min, while the 200 mg/kg dose produced the lowest numerical glucose values at 30, 60, 90, and 120 min after glucose administration. Based on the overall glycemic response, 200 mg/kg body weight was selected for both tinctures in the chronic diabetic experiment (Table 5).

### 2.5. Glycemic Evolution and Comparative Statistical Analysis

Streptozotocin administration (180 mg/kg body weight, intraperitoneally) induced a rapid, sustained, and progressively worsening hyperglycemic state in CD1 mice.

Baseline blood glucose levels (92.40 ± 10.36 mg/dL) increased to 335.00 ± 27.96 mg/dL at 72 h and further to 358.60 ± 27.44 mg/dL at day 35, demonstrating the stability and progression of the experimental diabetic model. Statistical comparison with the control group showed no significant differences at baseline (Mann–Whitney U = 12.000, Z = −0.105, *p* = 0.916), confirming initial homogeneity. From day 3 onward, highly significant differences were observed at all time points (U = 0.000, Z ≈ −2.611, *p* = 0.009; exact *p* = 0.008), confirming persistent hyperglycemia throughout the study.

In the control group treated with physiological saline, blood glucose values remained stable, ranging from 92.20 ± 6.76 mg/dL to 99.80 ± 8.35 mg/dL, with no statistically significant variation (*p* = 0.068), confirming metabolic stability.

In the group treated with *Vaccinium myrtillus*, baseline glycemia was 92.20 ± 12.52 mg/dL, increasing to 352.00 ± 22.37 mg/dL at 72 h and 328.80 ± 24.39 mg/dL at day 7. A progressive decrease was subsequently observed: 282.60 ± 14.74 mg/dL at day 14, 227.40 ± 17.50 mg/dL at day 21, 191.20 ± 16.18 mg/dL at day 28, and 174.80 ± 12.64 mg/dL at day 35.

Compared to the control group, glycemia remained significantly higher at all time points after induction (Mann–Whitney U = 0.000, Z ≈ −2.611, *p* = 0.009). However, comparison with the untreated diabetic group revealed a time-dependent antihyperglycemic effect. No statistically significant differences were observed at early time points (day 3: U = 8.000, Z = −0.940, *p* = 0.347; day 7: U = 9.500, Z = −0.629, *p* = 0.530). From day 14 onward, statistically significant reductions were observed (U = 0.000, Z ≈ −2.611, *p* = 0.009; exact *p* = 0.008), and this effect persisted through days 21, 28, and 35. At day 35, glycemia decreased to 174.80 mg/dL compared to 358.60 mg/dL in the untreated diabetic group, indicating a pronounced antihyperglycemic effect.

In the group treated with *Polygonum persicaria*, baseline glycemia was 89.40 ± 13.39 mg/dL, increasing to 420.80 ± 120.86 mg/dL at 72 h and reaching 481.60 ± 60.86 mg/dL at day 7. A gradual decrease was observed thereafter: 371.60 ± 119.71 mg/dL at day 14, 354.60 ± 116.73 mg/dL at day 21, 315.60 ± 100.31 mg/dL at day 28, and 263.50 ± 93.63 mg/dL at day 35 (*n* = 4, due to one mortality) (Table 6).

Compared to the control group, glycemia remained significantly elevated at all time points (Mann–Whitney U = 0.000, Z values between −2.611 and −2.449, *p* values between 0.009 and 0.014). When compared to the untreated diabetic group, no statistically significant differences were observed at most time points (day 3: U = 5.000, Z = −1.567, *p* = 0.117; day 14: U = 8.000, Z = −0.943, *p* = 0.346; day 21: U = 10.000, Z = −0.522, *p* = 0.602; day 28: U = 9.500, Z = −0.629, *p* = 0.530).

A statistically significant difference was observed at day 7 (U = 0.000, Z = −2.611, *p* = 0.009), where glycemia was higher than in the untreated group, indicating the absence of an early hypoglycemic effect. A statistically significant reduction compared to the untreated diabetic group was observed only at day 35 (U = 0.000, Z = −2.449, *p* = 0.014; exact *p* = 0.016), demonstrating a delayed onset of antihyperglycemic activity. All animals survived until the end of the experiment except one mouse in the *Polygonum persicaria* group; therefore, the final number of animals was *n* = 5 for the control, untreated diabetic, and *Vaccinium myrtillus* groups, and *n* = 4 for the *Polygonum persicaria* group.

### 2.6. Body Weight

In the control group, body weight showed a continuous increase throughout the experimental period, rising from 34.90 ± 2.79 g at baseline to 36.10 ± 2.46 g at 72 h, 37.30 ± 2.33 g at day 7, 38.50 ± 2.74 g at day 14, 40.00 ± 2.83 g at day 21, 41.40 ± 2.75 g at day 28, and reaching 42.80 ± 2.77 g at day 35. This trend reflects normal growth under physiological conditions.

In the untreated streptozotocin-induced diabetic group, body weight initially remained relatively stable (35.80 ± 1.30 g at baseline and 36.00 ± 2.12 g at 72 h), followed by a progressive decrease over time. Mean body weight declined to 33.70 ± 1.92 g at day 7, 31.30 ± 2.33 g at day 14, 30.10 ± 2.01 g at day 21, 29.30 ± 2.02 g at day 28, and reached 27.80 ± 1.82 g at day 35, indicating the catabolic effects associated with uncontrolled diabetes.

In the group treated with *Vaccinium myrtillus*, body weight showed a different pattern. After a slight decrease from 33.10 ± 1.19 g at baseline to 31.70 ± 2.11 g at 72 h, values stabilized and then progressively increased. Body weight reached 31.80 ± 2.17 g at day 7, 33.10 ± 1.60 g at day 14, 34.00 ± 1.37 g at day 21, 34.80 ± 1.04 g at day 28, and 36.20 ± 0.57 g at day 35. This trend indicates a recovery of body mass and suggests an improvement in metabolic status under treatment.

In the group treated with *Polygonum persicaria*, body weight exhibited fluctuations throughout the study. Initial values decreased from 30.90 ± 1.75 g at baseline to 30.10 ± 1.95 g at 72 h and further to 26.60 ± 4.34 g at day 7. Subsequently, a partial recovery was observed, with values increasing to 29.30 ± 2.91 g at day 14 and 31.20 ± 8.07 g at day 21. However, body weight decreased again to 28.60 ± 7.83 g at day 28 and 27.75 ± 7.32 g at day 35 (*n* = 4, due to one mortality).

### 2.7. Food and Water Intake

Food intake was significantly reduced in all diabetic groups compared to the control group (F = 10.793, *p* < 0.001). The control group showed the highest mean weekly food consumption (177.60 ± 8.14 g), whereas the untreated diabetic group had markedly lower values (100.40 ± 6.19 g).

Treatment with *Vaccinium myrtillus* (121.20 ± 7.56 g/week) and *Polygonum persicaria* (110.00 ± 24.24 g/week) did not restore food intake to control levels, both remaining significantly lower than the control (*p* < 0.001). However, when compared to the untreated diabetic group, no statistically significant differences were observed, indicating that neither treatment significantly modified feeding behavior in diabetic conditions.

Water intake was significantly increased in diabetic animals (F = 5.179, *p* = 0.001). The untreated STZ group exhibited pronounced polydipsia (326.00 ± 51.28 mL/week) compared to the control group (158.00 ± 16.43 mL/week).

The *Vaccinium myrtillus* group showed a lower mean water intake (215.00 ± 33.54 mL/week), approaching control values, although this reduction was not statistically significant compared to control or untreated diabetic animals. In contrast, the *Polygonum persicaria* group (290.00 ± 63.64 mL/week) maintained significantly higher water consumption than the control group (*p* = 0.012), indicating persistence of polydipsia.

### 2.8. Serum Lipid Profile

STZ-induced diabetes altered the serum lipid profile, increasing both total cholesterol and triglyceride levels compared with the non-diabetic control group. In the untreated diabetic group, total cholesterol increased from 158.00 ± 21.32 mg/dL at baseline to 186.40 ± 19.14 mg/dL at day 14 and remained elevated at day 35, reaching 183.80 ± 11.43 mg/dL. Triglyceride levels also increased from 138.00 ± 9.00 mg/dL at baseline to 191.80 ± 30.26 mg/dL at day 14 and 195.60 ± 28.06 mg/dL at day 35, indicating diabetes-associated dyslipidemia.

Treatment with *Vaccinium myrtillus* L. leaf tincture improved both lipid parameters by the end of the experiment. Total cholesterol decreased from 163.20 ± 12.03 mg/dL at day 14 to 139.40 ± 11.65 mg/dL at day 35, a value significantly lower than that recorded in the untreated diabetic group (*p* = 0.009). Triglycerides also decreased from 183.40 ± 9.32 mg/dL at day 14 to 150.40 ± 6.07 mg/dL at day 35, with a statistically significant reduction compared with the untreated diabetic group (*p* = 0.047).

In contrast, *Polygonum persicaria* L. herb tincture did not produce a significant improvement in the lipid profile. Total cholesterol remained elevated, with values of 204.40 ± 27.03 mg/dL at day 14 and 191.25 ± 36.43 mg/dL at day 35, without significant differences compared with the untreated diabetic group at day 35 (*p* = 1.000). Triglyceride levels were also increased at day 14, 224.20 ± 87.13 mg/dL, and remained high at day 35, 186.50 ± 85.34 mg/dL, with no statistically significant difference compared with the untreated diabetic group (*p* = 0.462) (Table 7).

These findings indicate a clear hypolipidemic effect for *Vaccinium myrtillus* L. leaf tincture, whereas *Polygonum persicaria* L. herb tincture showed no significant effect on serum cholesterol or triglycerides.

### 2.9. Oxidative Stress Parameters

Streptozotocin-induced diabetes was associated with a marked increase in oxidative stress, reflected by elevated MDA levels (mean 168.45) and reduced antioxidant enzyme activity, particularly GPx (mean 1410.40), compared to the control group (MDA mean 116.26; GPx mean 1869.60). These differences were statistically significant for MDA and GPx (*p* = 0.047), while TAC (mean 3.32 vs. 4.04) and SOD (mean 263.80 vs. 377.60) showed non-significant variations, indicating a partial impairment of antioxidant defense mechanisms.

Treatment with *Vaccinium myrtillus* resulted in a partial improvement of oxidative stress parameters. MDA levels decreased to a mean value of 155.51 compared to 168.45 in the untreated diabetic group, while SOD and GPx activities increased to mean values of 385.80 and 1799.80, respectively. Despite these favorable trends, none of these differences reached statistical significance (*p* > 0.05), suggesting a moderate but not statistically robust antioxidant effect.

In contrast, *Polygonum persicaria* demonstrated a more pronounced and selective antioxidant activity. GPx activity increased significantly to a mean value of 2473.67 compared to 1410.40 in the untreated diabetic group, with a statistically significant difference (*p* = 0.025), indicating strong activation of glutathione-dependent antioxidant pathways. At the same time, MDA levels decreased substantially to a mean value of 95.58 compared to 168.45, with a *p* value of 0.053, reflecting a clear trend toward statistical significance and a marked reduction in lipid peroxidation.

SOD activity increased to a mean value of 322.67 compared to 263.80 in the untreated diabetic group, but without statistical significance (*p* = 0.297), while TAC activity showed a slight increase to 3.87 compared to 3.32 (*p* = 0.456). These results suggest that *Polygonum persicaria* was associated with enhanced GPx activity and reduced lipid peroxidation under the present experimental conditions. However, because molecular regulators of antioxidant signaling were not assessed, these findings should be interpreted as functional evidence of increased glutathione-dependent antioxidant defense rather than proof of a specific GPx-mediated molecular pathway (Table 8).

When compared to the control group, GPx activity in the *Polygonum persicaria* group remained elevated, approaching statistical significance (*p* = 0.053), while SOD and TAC activities were slightly lower and MDA levels remained higher, indicating only partial normalization of oxidative stress.

### 2.10. Comparative Histopathological Analysis

Histological evaluation of the collected biological tissues aimed to highlight both the destructive effects induced by streptozotocin and the potential protective or adverse effects following the administration of plant-derived tinctures.

**Figure 8:**
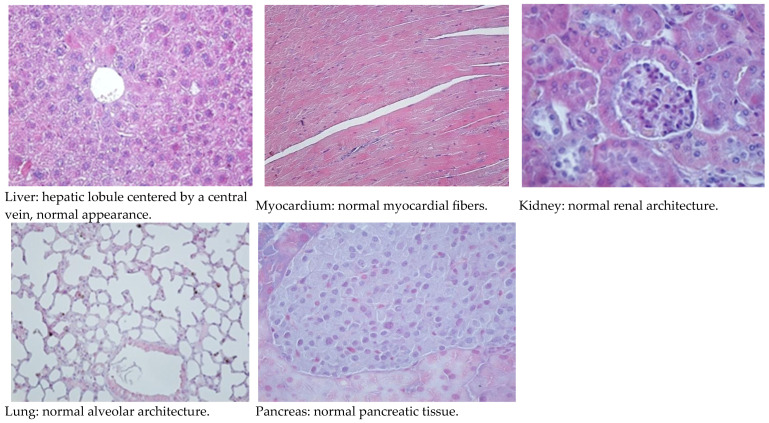
Representative histological sections from the healthy control group treated with physiological saline, showing preserved tissue architecture under routine hematoxylin–eosin staining: liver with preserved hepatic parenchyma, myocardium with organized muscle fibers, kidney with preserved glomerular and tubular structures, lung with preserved alveolar architecture, and pancreas showing a preserved islet-like endocrine structure surrounded by exocrine parenchyma.

**Figure 9:**
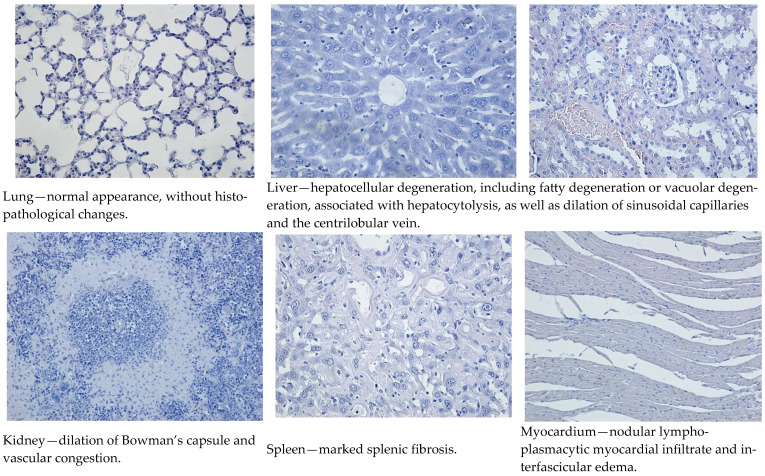
Histopathological aspects of organs collected from streptozotocin-induced diabetic mice treated with *Vaccinium myrtillus* L. leaf tincture. Lung with preserved architecture; liver showing fatty/vacuolar hepatocellular degeneration, hepatocytolysis, and dilation of sinusoidal capillaries and the centrilobular vein; kidney with Bowman’s capsule dilation and vascular congestion; spleen with marked fibrosis; myocardium with nodular lymphoplasmacytic infiltrate and interfascicular edema. Hematoxylin–eosin staining, ×100 magnification.

**Figure 10:**
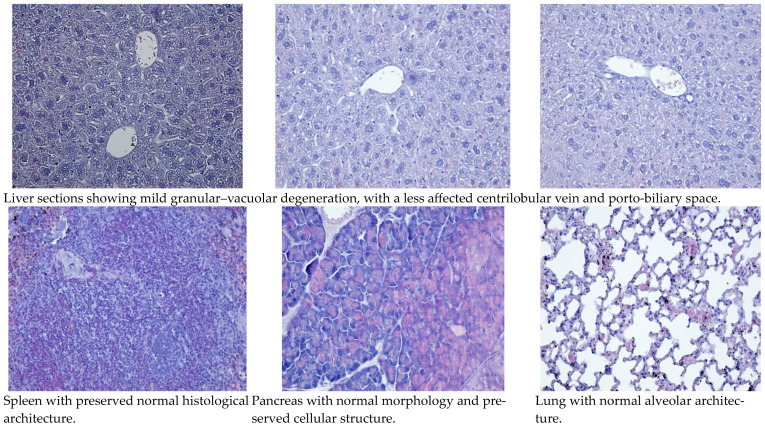
Histopathological aspects of organs collected from streptozotocin-induced diabetic mice treated with *Polygonum persicaria* L. herb tincture. Liver showing mild granular–vacuolar degeneration; spleen with preserved normal histological architecture; pancreas with preserved morphology and cellular structure; lung with normal alveolar architecture. Hematoxylin–eosin staining, ×100 magnification.

In the healthy control group treated with physiological saline, the examined organs showed preserved histological architecture under routine hematoxylin–eosin staining. The liver, myocardium, kidney, lung, and pancreas displayed normal structural organization, without obvious degenerative, inflammatory, congestive, or fibrotic alterations (Figure 8). The histopathological findings observed in the treated diabetic groups were therefore interpreted in comparison with this control morphology (Figure 8). In the group treated with *Vaccinium myrtillus*, multiple organs were affected, including the liver, kidney, myocardium, and spleen. Hepatic lesions were characterized by hepatocellular degeneration, including fatty and vacuolar changes, hepatocytolysis, and dilation of sinusoidal capillaries and the centrilobular vein. Renal alterations included dilation of Bowman’s capsule and vascular congestion, while myocardial tissue showed nodular lymphoplasmacytic infiltrates and interfascicular edema. Additionally, marked splenic fibrosis was observed. The pancreas could not be reliably evaluated in this group due to insufficient tissue preservation/availability; therefore, no definitive histopathological conclusion regarding pancreatic morphology could be drawn (Figure 9). In the group treated with *Polygonum persicaria*, only mild hepatic alterations were observed, consisting of slight granular–vacuolar degeneration, while the structure of the other organs remained largely preserved. This suggests a protective effect against streptozotocin-induced tissue damage (Figure 10).

*Polygonum persicaria* treatment demonstrated a predominantly protective histopathological profile, with tissue alterations limited mainly to mild hepatic changes, while preserving the structural integrity of most examined organs. This pattern suggests a relatively stable and non-disruptive biological effect, consistent with its predominantly antioxidant mechanism of action and limited metabolic interference. In contrast, *Vaccinium myrtillus* was associated with more pronounced and widespread structural changes affecting multiple organs, including the liver, kidney, myocardium, and spleen. These alterations, although indicative of tissue remodeling, should not be interpreted solely as adverse effects, but rather as part of a more complex biological response driven by its stronger metabolic activity and richer overall polyphenolic profile. Overall, these findings highlight a fundamental difference between the two extracts, with *Polygonum persicaria* exerting a more conservative and cytoprotective influence at the tissue level, whereas *Vaccinium myrtillus* induces a more dynamic and system-wide response involving both metabolic regulation and structural adaptation.

Compared with the healthy control group, which showed preserved organ architecture, the *Vaccinium myrtillus* group presented more heterogeneous structural alterations, whereas the *Polygonum persicaria* group showed a relatively more preserved histological profile, with only mild hepatic changes. However, these observations are based on routine hematoxylin–eosin staining and should be interpreted cautiously.

**Figure 11:**
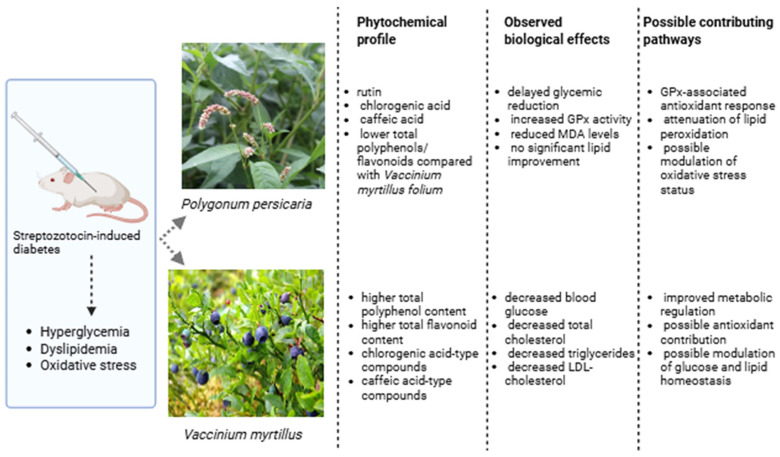
Schematic representation of the proposed antioxidant and metabolism-related pathways potentially involved in the effects of PPH and MF. MF may be associated with improved glycemic and lipid parameters, whereas PPH was mainly associated with antioxidant-related biochemical changes, including increased GPx activity and reduced lipid peroxidation. These mechanisms remain hypothetical and require confirmation by insulin measurements, pancreatic immunohistochemistry, and molecular analyses of antioxidant signaling pathways. Created with Biorender https://app.biorender.com/illustrations/canvas-beta/69c65d4b9962a1607b1eec2e (accessed on 1 June 2026).

To better summarize the comparative biological response patterns observed in the present study, a schematic figure was added to illustrate the proposed antioxidant- and metabolism-related pathways potentially associated with PPH and VMF treatment in streptozotocin-induced diabetic mice (Figure 11). This figure integrates the phytochemical profile, experimentally observed biological effects, and possible contributing pathways, while emphasizing that the proposed mechanisms require further molecular confirmation.

## 3. Discussion

The present study compared the antidiabetic, hypolipidemic, antioxidant, and histopathological effects of *Polygonum persicaria* and *Vaccinium myrtillus* in streptozotocin-induced diabetes. Overall, *Vaccinium myrtillus* showed a more pronounced antihyperglycemic and hypolipidemic effect, whereas *Polygonum persicaria* exhibited a delayed and modest glycemic effect without significant improvement in lipid parameters. Specifically, the reduction in glycemia induced by *Polygonum persicaria* reached statistical significance only at the end of the experimental period, while *Vaccinium myrtillus* produced a more rapid and sustained decrease in blood glucose levels [33,34]. In contrast, *Polygonum persicaria* was associated with an antioxidant-related biochemical response, mainly reflected by increased GPx activity and reduced lipid peroxidation.

The STZ-induced diabetic model used in this study is characterized by pancreatic β-cell injury, insulin deficiency, persistent hyperglycemia, weight loss, dyslipidemia, and oxidative stress [35,36,37]. These alterations were clearly observed in the untreated diabetic group and provided the experimental background for evaluating the two plant-derived preparations. However, because a standard antidiabetic drug control group was not included, the effects of the tinctures cannot be directly benchmarked against established pharmacological agents such as metformin or glibenclamide.

The phytochemical results showed differences in total polyphenol and flavonoid contents between the two tinctures. Nevertheless, the stronger antihyperglycemic and hypolipidemic effects observed for *Vaccinium myrtillus* cannot be attributed solely to these global phytochemical parameters. Total polyphenol and flavonoid contents do not fully capture qualitative differences in phytochemical composition, extraction efficiency, compound stability, bioavailability, metabolism, tissue distribution, or potential synergistic and antagonistic interactions among constituents. Therefore, the relationship between phytochemical profile and biological activity should be interpreted as associative rather than causal.

The antioxidant-associated profile observed for *Polygonum persicaria* should also be interpreted conservatively. Although increased GPx activity and reduced lipid peroxidation suggest an improvement in oxidative stress status, the present study did not assess circulating insulin levels, pancreatic β-cell preservation, or molecular antioxidant signaling pathways such as Nrf2/Keap1, HO-1, or NQO1. Consequently, the involvement of insulin-dependent mechanisms, β-cell protection, or specific redox signaling pathways remains speculative and requires further investigation. These findings are consistent with the broader concept that phenolic-rich plant extracts may influence oxidative stress and cytoprotective responses, although pathway-level confirmation requires dedicated molecular analyses [38,39].

Histopathological evaluation provided additional information on tissue-level changes. In the healthy control group treated with physiological saline, the examined organs showed preserved histological architecture under routine hematoxylin–eosin staining. Compared with this control morphology, *Vaccinium myrtillus* was associated with more heterogeneous structural alterations across several organs, including the liver, kidney, myocardium, and spleen. In contrast, *Polygonum persicaria* showed a relatively more preserved histological profile, with only mild hepatic changes and maintenance of the architecture in most examined organs.

However, routine hematoxylin–eosin staining alone does not allow definitive distinction between toxic injury, adaptive remodeling, inflammation, fibrosis, apoptosis, or repair. Therefore, the histopathological findings should be interpreted cautiously. Additional histochemical and immunohistochemical analyses, including Masson’s trichrome staining for fibrosis, TUNEL staining for apoptosis, and CD68/CD3 immunostaining for inflammatory cell infiltration, are required to clarify the biological significance of the observed tissue-level changes.

Several limitations should be acknowledged. The chronic pharmacodynamic experiment included a limited number of animals, with the *Polygonum persicaria* group reduced to *n* = 4 at the final time point. The STZ protocol used a single high dose, which may have induced a severe insulin-deficient diabetic state and limited the ability to detect moderate antihyperglycemic effects. In addition, circulating insulin levels, pancreatic immunohistochemistry, molecular antioxidant markers, and a complete dose–response evaluation were not included. HPTLC analysis was used mainly for comparative phytochemical screening and was not fully validated for definitive compound-specific quantification.

Future studies should include larger cohorts, prospective power analysis, multiple dose levels, standard antidiabetic drug controls, insulin measurements, pancreatic immunohistochemistry, validated HPLC-DAD or LC–MS phytochemical profiling, pharmacokinetic assessment, and molecular analyses of antioxidant and metabolic pathways. These approaches would help clarify whether the differences observed between *Polygonum persicaria* and *Vaccinium myrtillus* reflect true mechanistic divergence, differences in phytochemical composition and bioavailability, or different degrees of efficacy within the same experimental model.

## 4. Materials and Methods

### 4.1. Preparation of Tinctures by Simple Percolation (F.R. X)

The vegetal products *Polygoni persicariae herba* and Myrtilli folium were obtained from plant species grown in the “Alexandru Buia” University Botanical Garden, Craiova, Romania. The plant material was collected during the vegetation period, between May and September 2018. Botanical identification was performed within the institutional framework of the Botanical Garden and the Pharmacognosy Laboratory of the Faculty of Pharmacy, University of Medicine and Pharmacy of Craiova. Voucher/reference samples were deposited in the Collection of the Pharmacognosy Laboratory, Faculty of Pharmacy, University of Medicine and Pharmacy of Craiova, Romania. The plant materials were naturally dried, ground, and stored under appropriate conditions until extraction.

Tinctures were prepared by simple percolation according to the Romanian Pharmacopoeia (F.R. X), using a plant material/solvent ratio of 1:5 with 70% *(v/v*) ethanol. The plant materials, naturally dried and ground using an electric mill, were adjusted to the particle size corresponding to sieve IV. For moistening, 0.5 mL of diluted ethanol was added per gram of plant material, and the mixture was allowed to stand for 3 h at room temperature in tightly closed containers. The moistened material was subsequently passed through sieve I and introduced into the percolator with slight compression. The solvent was gradually added until it began to flow through the previously opened lower valve, leaving a thin liquid layer above the material. The valve was then closed, and the system was left to macerate for 24 h, after which percolation was initiated. The percolation rate was adjusted to obtain 1.5 g of extractive solution per gram of plant material within 24 h. Throughout the extraction period (two weeks), the plant material remained continuously covered with solvent. Percolation was stopped after obtaining 500 mL of each tincture. The tinctures were then stored for 7 days at 5–10 °C, filtered, and transferred into 100 mL amber glass bottles, tightly sealed and protected from light at room temperature [40,41,42].

### 4.2. HPTLC Screening of Flavonoids and Phenolcarboxylic Acids

High-performance thin-layer chromatography (HPTLC) was performed as a phytochemical screening method for the detection of flavonoids (flavonoid glycosides and corresponding aglycones) and phenolcarboxylic acids, including caffeic and chlorogenic acids, using appropriate reference standards [43,44,45,46]. The method is based on the differential migration of analytes on a silica gel stationary phase under the influence of a selective mobile phase. Identification was achieved by comparing the chromatographic bands of the samples with those of reference standards, followed by UV detection [47,48,49,50]. HPTLC analyses were performed on pre-coated silica gel 60 F254 glass plates (20 × 10 cm, Merck, Darmstadt, Germany). Plates were pre-washed with chloroform–methanol (1:1, *v/v*) and activated at 110 °C for 15 min. The mobile phase consisted of ethyl acetate–formic acid–methanol–water (15:1:0.1:1, *v/v/v/v*). Chromatographic development was carried out in a twin-trough chamber (20 × 10 cm) saturated with 10 mL mobile phase for 20 min (20 mL in the front channel and 10 mL in the rear channel).

Reference standards (Merck) included caffeic acid, chlorogenic acid, quercetin, and rutin, prepared as methanolic solutions (600 μg/mL). Samples consisted of 20% hydroalcoholic extracts. A volume of 2 μL of each standard and sample was applied as 8 mm bands, 8 mm from the lower edge of the plate, using a CAMAG Linomat 5 semi-automatic applicator (Muttenz, Switzerland) under nitrogen flow. Application parameters were: syringe volume 100 μL, application rate 150 nL/s, and pre-dosage volume 0.2 μL. The distance between tracks was 11.4 mm. Chromatographic development was performed up to a migration distance of 62 mm (solvent front at 70 mm), with an elution time of 13 min. After development, plates were dried for 3 min at 25 °C using cold air. Chromatographic plates were examined under UV light at 254 nm for visualization and documentation, and at 280 nm for densitometric analysis and in situ UV spectra acquisition. No derivatization was applied. Densitometric scanning was performed using a CAMAG TLC Scanner 3 controlled by visionCATS software (v. 2.5), with the following parameters: scanning speed 20 mm/s, resolution 100 μm/step, slit dimensions 5 × 0.2 mm, and detection in absorbance mode using deuterium and tungsten lamps.

The quantitative determination of phenylpropanoid compounds in tinctures was performed spectrophotometrically using the Arnow reagent, according to the Romanian Pharmacopoeia (F.R. IX, Cynarae folium monograph). The method is based on the formation of nitroso-derivatives through reaction with nitrous acid, followed by spontaneous isomerization to oximes, which exhibit a red coloration in alkaline medium. Quantification was carried out using a calibration curve constructed with caffeic acid standard solutions, measured at 510 nm (absorption maximum of caffeic acid oxime) using a JASCO V-530 UV–Vis spectrophotometer (JASCO Corporation, Tokyo, Japan),400–800 nm range. Absorbance values were maintained within 0.15–0.45 to comply with the Lambert–Beer law. The calibration equation was: y = 0.007922x + 0.01801 (R^2^ = 0.9873). Aliquots of 1 mL from each tincture were diluted to 10 mL with 70% ethanol. From these, 1 mL was transferred into 10 mL volumetric flasks, followed by the addition of 1 mL 0.5 M HCl, 1 mL Arnow reagent, and 1 mL 1 M NaOH, resulting in the formation of a red-colored complex. The solutions were brought to volume with 70% ethanol. Blank samples were prepared similarly, omitting the Arnow reagent. Absorbance was measured within 10 min at 510 nm using 1 cm quartz cuvettes, and results were expressed as the difference between sample and blank absorbance [51,52,53].

### 4.3. In Vitro Antioxidant Capacity Analysis

#### 4.3.1. Total Polyphenol Content

Total polyphenols were determined using the Folin–Ciocâlteu method [54,55,56,57]. A calibration curve was constructed using gallic acid (0–500 mg/L), with the equation y = 0.0038x + 0.2012 (R^2^ = 0.9722), measured at 765 nm. Tincture samples (diluted 1:5 with distilled water, 200 μL) were mixed with 2.5 mL Folin–Ciocâlteu reagent (1:10 dilution), followed after 4 min by 2 mL sodium carbonate solution (75 g/L). The mixtures were incubated for 2 h at room temperature (23 °C) in the dark. Absorbance was measured at 765 nm, and total polyphenol content was expressed as mg/L gallic acid equivalents (GAE).

#### 4.3.2. Total Flavonoid Content

Total flavonoids were determined spectrophotometrically using 10% aluminum chloride at 415 nm. A calibration curve was prepared using quercetin standard solutions (0–100 mg/L), yielding the equation y = 0.0093x − 0.0143 (R^2^ = 0.9843). Tincture samples (diluted 1:5) were mixed with 1.5 mL ethanol, 0.1 mL potassium acetate (10%), 0.1 mL aluminum chloride (10%), and 2.8 mL distilled water. After incubation at room temperature (23 °C) for 30 min, absorbance was measured at 415 nm. Results were expressed as mg/L quercetin equivalents (QE) [58,59,60].

### 4.4. Acute Toxicity Assessment in Mice

Acute oral toxicity was evaluated in CD1 male mice prior to the pharmacodynamic experiment, using an oral route of administration because this was the intended route for the subsequent in vivo study. The use of mice was selected in order to maintain consistency with the streptozotocin-induced diabetes model used in the pharmacological experiment. Animals were 6–8 weeks old, weighed 20–30 g, and were acclimatized for 7 days under standard laboratory conditions: temperature 22 ± 3 °C, relative humidity 30–70%, and a 12 h light/12 h dark cycle, with free access to food and water. The acute toxicity study was performed after a single oral administration of the hydroalcoholic tincture. The tested preparation was administered by oral gavage at increasing doses. In the first stage, doses of 1, 2, 3, 4, and 5 g/kg body weight were administered. Because no mortality was observed, a second stage was performed using higher doses of 6, 7, 8, and 9 g/kg body weight. Animals were observed continuously during the first hours after administration and then daily for 72 h. Mortality, behavioral changes, sedation, locomotor activity, respiratory changes, food and water intake, body weight, and other visible signs of toxicity were recorded. Body weight and blood glucose were measured before administration and 24 h after treatment. The median lethal dose (LD50) was estimated based on mortality after oral administration. Since no deaths occurred up to the highest tested dose of 9 g/kg body weight, the oral LD50 was considered to be higher than 9 g/kg body weight under the present experimental conditions [61]. Acute toxicity testing was performed only for *Polygonum persicaria* L. herb tincture because this preparation represented the less investigated vegetal product and the main object of the safety evaluation. In contrast, *Vaccinium myrtillus* L. leaf tincture was used as a positive phytotherapeutic comparator in the pharmacodynamic experiment, based on its previous experimental use and documented antidiabetic potential. The absence of a parallel acute toxicity test for *Vaccinium myrtillus* L. leaf tincture is acknowledged as a limitation of the present study.

### 4.5. Determination of Effective Dose Using Oral Glucose Tolerance Test (OGTT)

The oral glucose tolerance test was performed in male CD1 mice aged 4–6 weeks. Animals were divided into three experimental groups, with nine mice in each group. Group I served as the control group and received physiological saline. No conventional antidiabetic drug group, such as metformin or glibenclamide, was included in the present experimental design. Therefore, the study was designed to compare the biological effects of the two plant preparations with untreated diabetic animals and with a phytotherapeutic comparator, rather than to evaluate their efficacy relative to established clinical antidiabetic agents. Groups II and III received the tested hydroalcoholic tinctures and were each subdivided into three subgroups of three animals, corresponding to the three administered doses. The tested preparations were PPH tincture and MF tincture. Before glucose administration, animals were pretreated by oral gavage with the corresponding tincture at doses of 100, 150, or 200 mg/kg body weight. The highest dose, 200 mg/kg body weight, corresponded to one-tenth of the estimated LD50. The administered volume was 0.3 mL per animal. The control group received the same volume of physiological saline by oral gavage. Baseline blood glucose was measured after fasting. Thirty minutes after tincture or saline administration, glucose was administered orally at a dose of 2 g/kg body weight. Blood glucose levels were then measured at 30, 60, 90, and 120 min after glucose administration. All measurements were performed on the same day. Blood glucose was determined from tail-vein blood samples using an Accu-Chek Active glucometer. Results were expressed as mean ± standard deviation (SD) for each subgroup and were statistically compared with the control group.

### 4.6. Experimental Design and Glycemic Evaluation

The chronic pharmacodynamic experiment included 20 male CD1 mice aged 4–6 weeks, divided into four experimental groups of five animals each (*n* = 5/group). Animals were housed under standard laboratory conditions, with free access to standard chow and water, and were allowed to acclimatize before the beginning of the experiment. Group I served as the non-diabetic control group and received 0.9% saline solution. In Groups II–IV, type 1 diabetes mellitus was induced by a single intraperitoneal injection of streptozotocin (STZ) at a dose of 180 mg/kg body weight. Before STZ administration, animals were fasted for 12 h, with free access to water. Streptozotocin was freshly dissolved in cold 0.1 M citrate buffer, pH 4.5, immediately before intraperitoneal administration, because STZ rapidly degrades after dissolution. To prevent severe early post-STZ hypoglycemia, animals had access to 5% glucose solution for 24 h after STZ injection. Blood glucose was measured before STZ administration and again 72 h after STZ injection. Mice with fasting blood glucose values above 300 mg/dL, confirmed by repeated measurement, were considered diabetic and included in the chronic experiment. All animals included in the diabetic groups developed hyperglycemia after STZ administration. Group II served as the untreated diabetic control group and did not receive plant treatment. Group III served as the positive phytotherapeutic control and received *Vaccinium myrtillus* L. leaf hydroalcoholic tincture at a dose of 200 mg/kg body weight/day by oral gavage for 35 days. Group IV received *Polygonum persicaria* L. herb hydroalcoholic tincture at the same dose of 200 mg/kg body weight/day by oral gavage for 35 days. The dose of 200 mg/kg body weight/day was selected based on the preliminary oral glucose tolerance test, in which this dose produced the most favorable overall glycemic response for both tinctures. Hydroalcoholic tinctures were administered because they were prepared according to the Romanian Pharmacopoeia as traditional hydroalcoholic pharmaceutical preparations and because oral administration corresponds to the intended route for phytotherapeutic use. To minimize the possible influence of ethanol, the administered volume was kept constant and low. However, the absence of a separate ethanol vehicle-control group is acknowledged as a limitation of the study. Blood glucose levels were monitored throughout the 35-day experimental period using tail-vein blood samples and an Accu-Chek Active glucometer. At the end of the experiment, animals were fasted before blood collection and sacrifice. Blood samples were collected for biochemical analyses, while tissue samples were harvested for histopathological examination. All animals survived until the end of the experiment except one mouse in the *Polygonum persicaria* group. Therefore, the final number of animals was *n* = 5 for the non-diabetic control, untreated diabetic, and *Vaccinium myrtillus* groups, and *n* = 4 for the *Polygonum persicaria* group.

#### Animal Ethics

All animal experiments were conducted in accordance with relevant national, institutional, and international guidelines for the care and use of laboratory animals. The experimental protocol was reviewed and approved by the Ethics and Scientific Deontology Committee of the University of Medicine and Pharmacy of Craiova, Romania (Approval No. 09/28.03.2018). All efforts were made to minimize animal suffering and to reduce the number of animals used. The animal experiment was reported in accordance with the ARRIVE guidelines, and the completed ARRIVE checklist is provided as Supplementary Materials.

### 4.7. Histopathological Examination

At the end of the experimental period, mice were fasted overnight and deeply anesthetized with ketamine/xylazine, 100/10 mg/kg, intraperitoneally. Blood samples for biochemical analyses were collected immediately after anesthesia, and animals were subsequently euthanized for tissue harvesting. The pancreas, liver, kidney, spleen, myocardium, and lung were harvested, fixed in 10% neutral buffered formalin, dehydrated through graded alcohols, cleared in xylene, and embedded in paraffin.

### 4.8. Evaluation of Antioxidant Capacity in Chronic Experimental Diabetes

#### 4.8.1. MDA Assay

Malondialdehyde (MDA), a major end-product of lipid peroxidation, was used as a biomarker of oxidative stress. Reactive oxygen species induce peroxidation of polyunsaturated fatty acids, leading to the formation of lipid peroxides, which subsequently decompose into stable compounds, including MDA. Increased MDA levels reflect enhanced oxidative stress and impaired antioxidant status. MDA concentrations were determined using a commercially available ELISA kit (CEA597Ge; Cloud-Clone Corp., Katy, TX, USAsuitable for serum, plasma, and other biological fluids. The assay was performed using a complete analytical system (orbital shaker, washing system, and microplate reader; StatFax, Awareness Technology Inc., Palm City, FL, USA)) available in the Biochemistry Laboratory of the University of Medicine and Pharmacy of Craiova.

MDA levels in serum samples were determined using a competitive inhibition ELISA method based on microplates coated with a monoclonal anti-MDA antibody. Samples and standards were incubated with a biotin-labeled MDA conjugate, resulting in competition between labeled and unlabeled MDA for binding to the immobilized antibody. After incubation and washing, an avidin–peroxidase conjugate was added, which binds to the biotin-labeled complexes.

Following removal of unbound reagents, the enzyme-linked complex reacts with the substrate (tetramethylbenzidine, TMB), producing a blue-colored product. The reaction was stopped with an acidic solution, resulting in a yellow color, and absorbance was measured at 450 nm. The signal intensity is inversely proportional to the MDA concentration in the samples analyzed. Quantification was performed using a calibration curve generated from standard solutions. Blood samples were collected in EDTA tubes and centrifuged at 1000× *g* for 15 min at 4 °C within 30 min after collection. The obtained plasma was stored at −20 °C until biochemical analysis. Plasma malondialdehyde (MDA) levels were determined using a competitive ELISA method, according to the manufacturer’s instructions. Standards, controls, and plasma samples were analyzed in duplicate on 96-well plates pre-coated with anti-MDA antibody. The assay was based on the competition between sample MDA and MDA–biotin conjugate for antibody binding sites, followed by incubation with avidin–peroxidase conjugate and colorimetric detection using TMB substrate. The reaction was stopped with acidic stop solution, and absorbance was measured at 450 nm using a StatFax 2600 ELISA reader (Awareness Technology Inc., Palm City, FL, USA). A standard calibration curve was prepared using MDA standards ranging from 0 to 2000 ng/mL. Plasma MDA concentrations were calculated by interpolation from the calibration curve and expressed as ng/mL.

#### 4.8.2. Superoxide Dismutase (SOD) Activity

Erythrocyte superoxide dismutase (SOD) activity was determined using a commercial kit (Randox Laboratories Ltd., Crumlin, UK.) based on the generation of superoxide radicals (O_2_•^−^) through the xanthine–xanthine oxidase system. These radicals react with 2-(4-iodophenyl)-3-(4-nitrophenol)-5-phenyltetrazolium chloride (INT) to form a red formazan dye. SOD present in the sample competes with this reaction by catalyzing the dismutation of superoxide radicals into hydrogen peroxide and molecular oxygen. Therefore, SOD activity is measured as the degree of inhibition of formazan formation, with one unit of SOD defined as the amount causing 50% inhibition of the reaction rate. Hemolysates were obtained from whole blood by centrifugation, repeated washing of erythrocytes with saline, and subsequent hemolysis in cold distilled water. Samples were diluted with phosphate buffer (0.01 mol/L, pH 7) to achieve inhibition rates between 30% and 60%.

The reaction mixture contained substrate (xanthine and INT), buffer (CAPS, pH 10.2, with EDTA), and xanthine oxidase. Absorbance was measured at 505 nm at 30 s (A_1_) and after 3 min (A_2_), and the rate of reaction (ΔA/min) was calculated. SOD activity was expressed as U/mL whole blood based on calibration curves and dilution factors.

#### 4.8.3. Glutathione Peroxidase (GPx) Activity

Glutathione peroxidase (GPx) activity was determined using a Randox kit, (Randox Laboratories Ltd., Crumlin, UK) based on the enzymatic oxidation of reduced glutathione (GSH) by peroxides. In the presence of glutathione reductase (GR) and NADPH, oxidized glutathione (GSSG) is converted back to GSH, accompanied by oxidation of NADPH to NADP^+^. The decrease in absorbance at 340 nm, corresponding to NADPH consumption, is directly proportional to GPx activity. Whole blood samples were diluted with buffer and incubated with a reaction mixture containing glutathione, glutathione reductase, NADPH, and cumene hydroperoxide as substrate. Absorbance was measured at 340 nm at 1, 2, and 3 min intervals. GPx activity was calculated using the formula:

GPx activity (U/L hemolysate) = ΔA/min × 8412(1)

### 4.9. Total Antioxidant Capacity (TAC)

Total antioxidant capacity was determined using a Randox assay based on the generation of the ABTS^+^ radical cation (2,2′-azino-di-[3-ethylbenzothiazoline sulfonate]) through the reaction of ABTS with methemoglobin and hydrogen peroxide. The resulting blue-green chromophore exhibits maximum absorbance at 600 nm. Antioxidants present in the sample inhibit the formation of this radical, and the decrease in color intensity is inversely proportional to the antioxidant capacity. Serum samples, standards, and blanks were mixed with chromogen reagent and incubated at 37 °C. Initial absorbance (A_1_) was recorded, followed by the addition of hydrogen peroxide substrate and measurement of absorbance after 3 min (A_2_). The change in absorbance (ΔA) was used to calculate antioxidant capacity using a calibration factor derived from the standard:

TAC (mmol/L) = F × (ΔA_blank_ − ΔA__sample_) (2)

### 4.10. Statistical Analysis

Statistical analysis was performed using IBM SPSS Statistics software (version 23). Descriptive analysis of the study groups included the calculation of mean values, minimum and maximum values, and standard deviation. Comparative analysis was conducted using nonparametric tests, with statistical significance set at *p* < 0.05 (95% confidence level). The Mann–Whitney U test was applied for comparisons between independent groups (e.g., blood glucose levels between different experimental groups), while the Wilcoxon signed-rank test was used for paired comparisons within the same group (e.g., blood glucose levels at day 1 versus day 7). Given the limited number of animals included in each experimental group, the statistical analysis was interpreted cautiously. Nonparametric tests were selected because they are suitable for small sample sizes and do not require normal distribution assumptions. Nevertheless, the relatively small sample size, particularly the reduction in the *Polygonum persicaria* group to *n* = 4 at day 35 due to one mortality, may increase the risk of both type I and type II statistical errors. Therefore, the present findings should be regarded as preliminary and exploratory, and confirmation in larger, prospectively powered studies is required.

## 5. Conclusions

The present study demonstrates that *Polygonum persicaria* and *Vaccinium myrtillus* exert distinct biological effects in streptozotocin-induced diabetes. *Vaccinium myrtillus* showed a pronounced and statistically significant antihyperglycemic and hypolipidemic activity, whereas *Polygonum persicaria* exhibited a delayed and modest glycemic effect, reaching statistical significance only at the end of the experimental period (*p* = 0.014), without significant improvement in lipid parameters.

In contrast, *Polygonum persicaria* displayed a selective antioxidant profile, evidenced by a significant increase in GPx activity (*p* = 0.025) and a reduction in MDA levels approaching statistical significance (*p* = 0.053), suggesting attenuation of lipid peroxidation. However, because circulating insulin levels, pancreatic β-cell markers, and molecular antioxidant signaling pathways were not assessed, the underlying mechanisms cannot be definitively established. Therefore, these findings should be interpreted as evidence of an antioxidant-associated biochemical response rather than proof of a specific mechanism of action.

Phytochemical analysis revealed lower total polyphenol and flavonoid contents in *Polygonum persicaria* compared with *Vaccinium myrtillus*. However, these global parameters cannot fully explain the differences in biological activity observed between the two extracts. Qualitative phytochemical composition, extraction efficiency, compound stability, bioavailability, metabolism, tissue distribution, and interactions among constituents may also have contributed to the observed effects. Therefore, the association between the richer overall phytochemical profile of *Vaccinium myrtillus* and its broader biological activity should be interpreted cautiously and not as a direct causal relationship. Overall, under the present experimental conditions, *Polygonum persicaria* showed limited antihyperglycemic activity and was associated with an antioxidant-related biochemical response. This pattern should not be interpreted as proof of a selective mechanism, because it may also reflect insufficient metabolic efficacy in the present experimental model. Therefore, it cannot be considered a primary antidiabetic agent based on the current data, although it may warrant further investigation as a potential adjunctive strategy targeting oxidative stress in diabetes. These conclusions should be interpreted with caution because of the exploratory design and the limited sample size, especially for the *Polygonum persicaria* group at the final time point.

Further studies including larger cohorts, prospective power analysis, and additional mechanistic biomarkers are necessary to confirm the reproducibility and translational relevance of these findings. In particular, future investigations should include validated HPLC-DAD or LC–MS phytochemical profiling, multiple-dose pharmacodynamic evaluation, insulin measurements, pancreatic immunohistochemistry, and molecular analyses of antioxidant signaling pathways. Moreover, the inclusion of a standard antidiabetic drug control group, such as metformin or glibenclamide, would allow direct comparison with established pharmacological therapy and help define the therapeutic relevance of these plant extracts more precisely.

## Figures and Tables

**Table 1:** Results obtained from the HPTLC analysis of polyphenols in the PPH tincture. CSS: Thin-layer chromatography; PPH: *Polygoni persicariae herba*; AU: Area units.

Peak No.	Rf Max.	AU	Area [%]	Observations
1.	0.006	0.018890581	13.92	HPTLC analysis of the *Polygonum persicaria* tincture detected several chromatographic bands based on retention factor (Rf) values and comparison with available reference standards. A band corresponding to rutin was observed at Rf = 0.11, while bands corresponding to chlorogenic acid and caffeic acid were observed at Rf = 0.29 and Rf = 0.88, respectively. The associated densitometric responses are reported only as semi-quantitative comparative estimates and should not be interpreted as validated concentrations.
2.	0.065	0.008773567	6.47	
3.	0.089	0.004032056	2.97	
4.	0.11	0.005787209	4.27	
5.	0.17	0.00525282	3.87	
6.	0.21	0.004494693	3.31	
7.	0.29	0.007883445	5.83	
8.	0.33	0.006631081	4.89	
9.	0.38	0.007394359	5.45	
10.	0.44	0.018953541	13.97	
11.	0.54	0.009298141	6.85	
12.	0.62	0.003906682	2.88	
13.	0.69	0.004513911	3.33	
14.	0.83	0.015386476	11.34	
15.	0.88	0.006178941	4.55	
16.	0.92	0.004278723	3.15	
17.	0.94	0.004003793	2.95	

**Table 2:** Results obtained from the HPTLC analysis of polyphenols in the MF tincture. CSS: Thin-layer chromatography; MF: *Myrtilli folium*; AU: Area units.

Peak No.	Rf Max.	AU	Area [%]	Observations
1.	0.006	0.028906266	9.65	HPTLC analysis of the MF tincture revealed several phenolcarboxylic acid-type chromatographic bands identified based on retention factor (Rf) values and comparison with available reference standards. Bands corresponding to chlorogenic acid and caffeic acid were observed at Rf = 0.29 and Rf = 0.89, respectively. The stronger densitometric response suggests a higher relative abundance of these marker compounds in MF; however, these data are semi-quantitative comparative estimates and not validated concentrations.
2.	0.07	0.016490434	5.5	
3.	0.15	0.013662637	4.56	
4.	0.24	0.03340745	11.15	
5.	0.29	0.047202027	15.75	
6.	0.38	0.036308042	12.12	
7.	0.47	0.032615418	10.88	
8.	0.55	0.010092842	3.37	
9.	0.61	0.013189721	4.4	
10.	0.67	0.007858997	2.62	
11.	0.73	0.005794021	1.93	
12.	0.84	0.014537034	4.85	
13.	0.89	0.018878335	6.3	
14.	0.92	0.020741539	6.92	

**Table 3:** In vitro antioxidant activity of hypoglycemic tinctures. GAE: gallic acid equivalents; EQ: quercetin equivalents.

Tinctures	In Vitro Antioxidant Activity	
	Total Polyphenol Content [mg/L GAE]	Total Flavonoid Content [mg/L QE]
PPH	269.28 ± 5.25	132.75 ± 2.65
MF	433.89 ± 8.67	154.38 ± 3.08

**Table 4:** Determination of acute toxicity for the *Polygonum persicaria* tincture at doses of 6, 7, 8, and 9 g/kg body weight.

Mouse	Tincture Dose	Body Weight Before Administration (g)	Body Weight at 24 h (g)	Blood Glucose Before Administration (mg/dL)	Blood Glucose at 24 h (mg/dL)
1.	6 g/kg bw	31	29.5	103	111
2.	6 g/kg bw	29.5	27.5	74	108
3.	7 g/kg bw	33	32	87	93
4.	7 g/kg bw	27.5	26.5	51	81
5.	8 g/kg bw	26.5	26	70	121
6.	8 g/kg bw	33	32	91	46
7.	9 g/kg bw	32.5	31	81	102
8.	9 g/kg bw	34	33.5	94	107

**Table 5:** Effect of *Polygonum persicaria* L. herb and *Vaccinium myrtillus* L. leaf tinctures on blood glucose levels during the oral glucose tolerance test.

	Group	Fasting	30 min	60 min	90 min	120 min
Control		158 ± 27.87	147.33 ± 22.68	142 ± 15.62	150 ± 7.21	137.67 ± 3.79
*Polygonum persicaria*	100 mg/kg bw	132.67 ± 5.13	146.33 ± 32.81	155.67 ± 36.83	143 ± 38	134.67 ± 31.66
	150 mg/kg bw	125 ± 16	138 ± 33.42	131.33 ± 26.03	123 ± 20.0	126.33 ± 19.43
	200 mg/kg bw	125 ± 27.87	121.67 ± 28.01	123.67 ± 15.52	122 ± 13.11 *	122 ± 1.73 *
*Vaccinium myrtillus*	100 mg/kg bw	134 ± 15.62	185.33 ± 56.07	161 ± 30.45	136.33 ± 19.14	114.67 ± 7.37 *
	150 mg/kg bw	122 ± 12.53	175.33 ± 49.17	154 ± 19.31	146.33 ± 16.65	120.67 ± 3.51 *
	200 mg/kg bw	120.67 ± 14.47	137.33 ± 28.22	126 ± 23.3	112 ± 22.61	101 ± 18.36

Values are expressed as mean ± SD; * *p* < 0.05 versus control.

**Table 6:** Evolution of blood glucose levels in experimental groups during the 35-day experiment.

Group	Baseline	72 h After STZ	Day 7	Day 14	Day 21	Day 28	Day 35
Control	92.20 ± 6.76	93.60 ± 4.39	95.00 ± 4.18	99.80 ± 10.06	97.20 ± 11.01	97.80 ± 9.73	99.80 ± 8.35
Untreated diabetic	92.40 ± 10.36	335.00 ± 27.96	341.20 ± 30.82	349.00 ± 29.92	352.40 ± 29.74	355.00 ± 28.83	358.60 ± 27.44
*Vaccinium myrtillus* L. leaf tincture	92.20 ± 12.52	352.00 ± 22.37	328.80 ± 24.39	282.60 ± 14.74	227.40 ± 17.50	191.20 ± 16.18	174.80 ± 12.64
*Polygonum persicaria* L. herb tincture	89.40 ± 13.39	420.80 ± 120.86	481.60 ± 60.86	371.60 ± 119.71	354.60 ± 116.73	315.60 ± 100.31	263.50 ± 93.63

Values are expressed as mean ± SD. For the *Polygonum persicaria* group, *n* = 5 from baseline to day 28 and *n* = 4 at day 35 due to one mortality. STZ: streptozotocin.

**Table 7:** Serum lipid profile in experimental groups.

Group	Total Cholesterol Baseline	Total Cholesterol Day 14	Total Cholesterol Day 35	Triglycerides Baseline	Triglycerides Day 14	Triglycerides Day 35
Control	82.40 ± 7.02	90.60 ± 9.29	93.60 ± 8.50	125.20 ± 16.30	131.60 ± 17.27	133.20 ± 17.24
Untreated diabetic	158.00 ± 21.32	186.40 ± 19.14	183.80 ± 11.43	138.00 ± 9.00	191.80 ± 30.26	195.60 ± 28.06
*Vaccinium myrtillus* L. leaf tincture	136.20 ± 9.60	163.20 ± 12.03	139.40 ± 11.65	94.40 ± 22.66	183.40 ± 9.32	150.40 ± 6.07
*Polygonum persicaria* L. herb tincture	131.40 ± 7.89	204.40 ± 27.03	191.25 ± 36.43	95.00 ± 30.01	224.20 ± 87.13	186.50 ± 85.34

Values are expressed as mean ± SD. For the *Polygonum persicaria* group, *n* = 5 at baseline and day 14 and *n* = 4 at day 35. Lipid parameters are expressed as mg/dL.

**Table 8:** Oxidative stress biomarkers in experimental groups.

Group	MDA	TAC	SOD	GPx
Control	116.26 ± 32.55	4.04 ± 0.49	377.60 ± 103.00	1869.60 ± 154.54
Untreated diabetic	168.45 ± 41.82	3.32 ± 0.79	263.80 ± 70.34	1410.40 ± 395.98
*Vaccinium myrtillus* L. leaf tincture	155.51	3.98 ± 0.57	385.80 ± 100.44	1799.80 ± 499.14
*Polygonum persicaria* L. herb tincture	95.58	3.87	322.67	2473.67

Values are expressed as mean ± SD where available. MDA: malondialdehyde; TAC: total antioxidant capacity; SOD: superoxide dismutase; GPx: glutathione peroxidase.

## Data Availability

To promote transparency and reproducibility, we will provide a detailed data availability statement. The files and data are in the physical and electronic archive of the University of Medicine and Pharmacy Craiova and can be requested from the corresponding author. The original contributions presented in this study are included in the article. Further inquiries can be directed to the corresponding authors.

## Supplementary Materials

The following supporting information can be downloaded at: https://www.mdpi.com/article/10.3390/molecules31122080/s1. The ARRIVE guidelines 2.0: author checklist.
