# Supplementary material for: Comparative Evaluation of the Antidiabetic, Hypolipidemic and Antioxidant Effects of Polygonum persicaria L. Herb and Vaccinium myrtillus L. Leaves in Streptozotocin-Induced Diabetes
Source: Molecules. 2026 Jun 13;31(12):2080. doi: 10.3390/molecules31122080 (PMC13304656; doi:10.3390/molecules31122080)
Supplement: Supplementary file 1 [file molecules-31-02080-s001.zip › molecules-4357005-supplementary.pdf]

NOTE: Please save this file locally before filling in the table, DO NOT work on the file within your internet browser as changes will not be saved. Adobe Acrobat Reader (available free here) is recommended for completion.

The ARRIVE guidelines 2.0: author checklist

The ARRIVE Essential 10

These items are the basic minimum to include in a manuscript. Without this information, readers and reviewers cannot assess the reliability of the findings.

Item		Recommendation	Section/line number, or reason for not reporting
**Study design**	1	For each experiment, provide brief details of study design including:<br>a. The groups being compared, including control groups. If no control group has been used, the rationale should be stated.<br>b. The experimental unit (e.g. a single animal, litter, or cage of animals).	Materials and Methods 4.5.3<br>Four experimental groups were included: a normal control group (Group I), a diabetic control group (Group II), and two treatment groups receiving Acanthus balcanicus (Group III) and Vaccinium myrtillus (Group IV). The
**Sample size**	2	a. Specify the exact number of experimental units allocated to each group, and the total number in each experiment. Also indicate the total number of animals used.<br>b. Explain how the sample size was decided. Provide details of any *a priori* sample size calculation, if done.	Materials and Methods 4.5.3<br>Each group consisted of 5 animals (total 20). The sample size was determined based on previous similar experimental studies; no formal a priori sample size calculation was performed.
**Inclusion and exclusion criteria**	3	a. Describe any criteria used for including and excluding animals (or experimental units) during the experiment, and data points during the analysis. Specify if these criteria were established *a priori*. If no criteria were set, state this explicitly.<br>b. For each experimental group, report any animals, experimental units or data points not included in the analysis and explain why. If there were no exclusions, state so.<br>c. For each analysis, report the exact value of *n* in each experimental group.	Materials and Methods 4.5.2<br>Animals with fasting blood glucose levels exceeding 290–310 mg/dL at 72 h and 1 week post streptozotocin administration were included as diabetic. No additional exclusion criteria were applied. No animals or data [illegible]
**Randomisation**	4	a. State whether randomisation was used to allocate experimental units to control and treatment groups. If done, provide the method used to generate the randomisation sequence.<br>b. Describe the strategy used to minimise potential confounders such as the order of treatments and measurements, or animal/cage location. If confounders were not controlled, state this explicitly.	Animals were randomly allocated to experimental groups; however, the method of randomisation was not formally recorded.
**Blinding**	5	Describe who was aware of the group allocation at the different stages of the experiment (during the allocation, the conduct of the experiment, the outcome assessment, and the data analysis).	No blinding was performed during allocation, experimental procedures, outcome assessment, or data analysis.
**Outcome measures**	6	a. Clearly define all outcome measures assessed (e.g. cell death, molecular markers, or behavioural changes).<br>b. For hypothesis-testing studies, specify the primary outcome measure, i.e. the outcome measure that was used to determine the sample size.	Methods + Results<br>Outcome measures included blood glucose levels, lipid profile (cholesterol and triglycerides), body weight, food and water intake, and oxidative stress markers (SOD, GPx, GR, lipid peroxidation).<br>The primary outcome measure was fasting
**Statistical methods**	7	a. Provide details of the statistical methods used for each analysis, including software used.<br>b. Describe any methods used to assess whether the data met the assumptions of the statistical approach, and what was done if the assumptions were not met.	4.5.5 Statistical Analysis<br>Data were analyzed using Student's t test. Statistical significance was set at $p < 0.05$. No formal assessment of normality or variance homogeneity was performed.
**Experimental animals**	8	a. Provide species-appropriate details of the animals used, including species, strain and substrain, sex, age or developmental stage, and, if relevant, weight.<br>b. Provide further relevant information on the provenance of animals, health/immune status, genetic modification status, genotype, and any previous procedures.	Male Swiss albino mice, 6–8 weeks old, weighing 35–45 g, were used. Animals were obtained from the institutional biobase and acclimatized for one week prior to the experiment.
**Experimental procedures**	9	For each experimental group, including controls, describe the procedures in enough detail to allow others to replicate them, including:<br>a. What was done, how it was done and what was used.<br>b. When and how often.<br>c. Where (including detail of any acclimatisation periods).<br>d. Why (provide rationale for procedures).	Streptozotocin was administered intraperitoneally at 180 mg/kg. Plant extracts were administered orally (145 mg/kg) once daily for 5 weeks. Measurements were performed weekly after 12 h fasting
**Results**	10	For each experiment conducted, including independent replications, report:<br>a. Summary/descriptive statistics for each experimental group, with a measure of variability where applicable (e.g. mean and SD, or median and range).<br>b. If applicable, the effect size with a confidence interval.	Results are reported as mean ± standard deviation. Significant differences between groups were identified using statistical testing and trends were presented in tables (Tables 1–7).

The Recommended Set

These items complement the Essential 10 and add important context to the study. Reporting the items in both sets represents best practice.

Item		Recommendation	Section/line number, or reason for not reporting
**Abstract**	11	Provide an accurate summary of the research objectives, animal species, strain and sex, key methods, principal findings, and study conclusions.	Reported in Abstract section.
**Background**	12	a. Include sufficient scientific background to understand the rationale and context for the study, and explain the experimental approach.<br>b. Explain how the animal species and model used address the scientific objectives and, where appropriate, the relevance to human biology.	Introduction
**Objectives**	13	Clearly describe the research question, research objectives and, where appropriate, specific hypotheses being tested.	The objective was to compare the phytochemi
**Ethical statement**	14	Provide the name of the ethical review committee or equivalent that has approved the use of animals in this study, and any relevant licence or protocol numbers (if applicable). If ethical approval was not sought or granted, provide a justification.	Approved by Ethics Committee (Approval No. 09/28.03.2018).
**Housing and husbandry**	15	Provide details of housing and husbandry conditions, including any environmental enrichment.	Methods
**Animal care and monitoring**	16	a. Describe any interventions or steps taken in the experimental protocols to reduce pain, suffering and distress.<br>b. Report any expected or unexpected adverse events.<br>c. Describe the humane endpoints established for the study, the signs that were monitored and the frequency of monitoring. If the study did not have humane endpoints, state this.	Animals were monitored daily. A 5% glucose solution was administered post streptozotocin to prevent hypoglycemia. No adverse events were observed.
**Interpretation/ scientific implications**	17	a. Interpret the results, taking into account the study objectives and hypotheses, current theory and other relevant studies in the literature.<br>b. Comment on the study limitations including potential sources of bias, limitations of the animal model, and imprecision associated with the results.	Discussion
**Generalisability/ translation**	18	Comment on whether, and how, the findings of this study are likely to generalise to other species or experimental conditions, including any relevance to human biology (where appropriate).	Findings suggest potential relevance to human diabetes; however, translation is limited due to the use of an animal model.
**Protocol registration**	19	Provide a statement indicating whether a protocol (including the research question, key design features, and analysis plan) was prepared before the study, and if and where this protocol was registered.	No protocol was registered prior to the study.
**Data access**	20	Provide a statement describing if and where study data are available.	Reported in Data Availability Statement sectio
**Declaration of interests**	21	a. Declare any potential conflicts of interest, including financial and non-financial. If none exist, this should be stated.<br>b. List all funding sources (including grant identifier) and the role of the funder(s) in the design, analysis and reporting of the study.	Reported in Conflicts of Interest and Funding sections.<br>The authors declare no conflicts of interest.<br>The study received no external funding.
